# Associations between Chronic Pain and Attention-Deficit Hyperactivity Disorder (ADHD) in Youth: A Scoping Review

**DOI:** 10.3390/children10010142

**Published:** 2023-01-11

**Authors:** Eleanor A. J. Battison, Patrick C. M. Brown, Amy L. Holley, Anna C. Wilson

**Affiliations:** 1 Oregon Health & Science University School of Medicine, Portland, OR 97239, USA; 2 Department of Pediatrics, Oregon Health & Science University, Portland, OR 97239, USA

**Keywords:** chronic pain, ADHD, youth, pediatric

## Abstract

**Background:** Chronic pain and ADHD are common conditions among youth that negatively impact functioning. This review fills a critical gap by summarizing current research on chronic pain and ADHD comorbidity, and it proposes a conceptual model of shared associations and underlying mechanisms. **Objective:** The aims of the current study were to: (1) review the extant literature and present estimates of the prevalence of comorbid non-headache chronic pain and ADHD in youth and (2) describe potential shared mechanisms for ADHD and chronic non-headache pain in youth. We also outline future directions to inform future research and interventions directed to youth with comorbid pain and ADHD. **Design:** A scoping review of the literature was performed in MEDLINE, PsycInfo, and Cochrane Database of Systematic Reviews using a wide range of search terms related to pain, Attention Deficit-Hyperactivity Disorder, childhood, adolescence, and young adulthood. **Results:** Eleven published studies were included in the review. These studies examined the prevalence of chronic pain among youth with ADHD, the prevalence of ADHD in chronic pain samples, and the association between chronic pain and ADHD among youth. Findings revealed results from studies indicating a higher prevalence of ADHD among youth with chronic pain and a higher prevalence of chronic pain in samples of youth with ADHD. **Conclusions:** Findings from this scoping review suggest an association between chronic pain and ADHD among youth. Little research was found to examine the etiology of this association. Future studies should examine underlying mechanisms of comorbid chronic pain and ADHD.

## 1. Introduction

Chronic pain, defined as pain lasting 3 months or longer [1], is common during childhood. Approximately 23% of children and adolescents experience chronic pain [2], and it has a significant impact on psychological, social and economic outcomes [3,4]. The etiology of chronic pain varies, and in 13–33% of cases, there is no known etiology to chronic pain in youth [5,6]. Chronic pain is associated with significant disability as well as poor outcomes that persist into adulthood [7]. Youth with chronic pain are more likely to report poor quality of life, problems with social and school functioning, and poorer mental health [8]. Furthermore, female sex, depression, poor sleep, post-traumatic stress and parental factors [9] are risk factors for developing chronic pain in youth [10,11]. However, the mechanisms by which these risk factors lead to the development of chronic pain among youth are not yet well understood. 

ADHD is a neurodevelopmental disorder that impairs cognitive, emotional, and behavioral functioning. It is characterized by either attentional problems (inattentive presentation), hyperactivity (hyperactive presentation), or most commonly both (combined presentation) [12]. Prevalence estimates of ADHD vary depending on country and study methodology, but it affects approximately 3–4% of children and adolescents worldwide [13,14]. Prevalence estimates vary primarily due to year of study, country of origin and diagnostic criteria. The etiology of ADHD is complex and not well understood, but evidence supports theories of delayed brain maturation and dopamine system dysfunction [15,16]. ADHD in childhood and adolescence is associated with a range of adverse health outcomes, including poor academic achievement, social problems, comorbid psychopathology and functional impairment [17], which often persist into adulthood [18]. Untreated ADHD in adulthood is associated with poor quality of life and high risk of substance use [19]. Furthermore, the societal and economic impacts of ADHD are significant across the lifespan [20]. Additional research is needed to investigate etiologies of ADHD and more effective treatment of ADHD.

### Chronic Pain and ADHD

Research on pediatric chronic pain suggests comorbidity and related mechanisms with a variety of psychiatric disorders, including depression [21], anxiety disorders [22] and post-traumatic stress disorder (PTSD) [23]. Many of these psychological factors function as risk factors for chronic pain [24]. However, only a few studies have examined the prevalence of ADHD and comorbid chronic pain, and the majority of existing research has been conducted with adults [15]. A 2018 systematic review by Instanes et al. focused on associations between adult ADHD and comorbid medical/somatic diseases [25]. The review found increased prevalence rates of various somatic diseases but did not specifically focus on pain-related conditions. Other studies with adults have found that ADHD is more prevalent among people with various chronic pain conditions [26,27,28,29,30]. Retrospective studies have found that childhood ADHD is associated with higher rates of fibromyalgia in adulthood [31,32]. Moreover, research suggests that ADHD is associated with increased pain perception [33], that chronic pain reduces attention span [30], and that adults with ADHD have significantly higher prevalence of pain reports compared to controls [26]. Similarly, prevalence studies suggest that children with hyperactivity/inattention problems are more likely to experience multiple, recurrent subjective health complaints [34]. Recent reviews of the pediatric literature have also found that ADHD is associated with migraines [35] and other headaches [36]. Prevalence rates of ADHD were found to range between 36.5 and 20% for children diagnosed with primary headache disorders, tension type headaches and migraines. Additionally, ADHD has been suggested as a risk factor for sensory processing disorders and increased prevalence of neurologic disorders among children [37,38]. These conditions might in turn affect the pain experience and possibly the development of chronic pain.

The potential association between ADHD and pediatric chronic non-headache pain has not yet been systematically reviewed. The aims of the current study were to: (1) review the extant literature and present estimates of the prevalence of comorbid non-headache chronic pain and ADHD in youth and (2) describe potential shared mechanisms for ADHD and non-headache chronic pain in youth. We also outline future directions to inform future research and interventions directed to youth with comorbid pain and ADHD. 

## 2. Aim 1: Scoping Review of the Literature

A substantial body of research has demonstrated associations among youth with chronic pain and internalizing symptoms (e.g., depression [21] and anxiety [22]), but there has been less focus on associations between chronic pain and ADHD. Additionally, little research has focused on children with ADHD and their experience with chronic pain. Given the availability of a recently published systematic review on ADHD and headache in childhood [36], we performed a scoping review to examine the association between ADHD and non-headache chronic pain to help identify gaps in the extant research that may warrant further investigation. 

### 2.1. Methods

This scoping review was conducted between November 2020 and April 2022. The authors created an unregistered search and review protocol based on the methods practices of the Joanna Briggs Institute Manual of Evidence Synthesis [39], and they utilized the framework described by Arksey and O’Malley [40]. MEDLINE, PsycInfo, and the Cochrane Database of Systematic Reviews were searched using a wide range of terms related to pain, Attention Deficit-Hyperactivity Disorder, childhood, adolescence, and young adulthood. Search terms were developed in consultation with an academic librarian. Mean sample ages up to 25 were included in the review because adult samples studying these research questions typically focus on ages 25–64 [41]. The search strategy is detailed in Supplementary Materials. The latest search occurred on 18 January 2022. Duplicates were deleted. Bibliographies of reviews identified during the initial search and articles included were searched for additional articles that met eligibility criteria.

#### 2.1.1. Eligibility Criteria

Inclusion and exclusion criteria were determined by the study team based on the guiding research and an initial assessment of the existing literature. Abstracts of scholarly journal articles were reviewed according to the following inclusion criteria: (1) description of ADHD prevalence in a chronic pain sample, chronic pain prevalence in an ADHD sample, or an analysis of the association between ADHD and chronic pain, (2) mean sample age between 3 and 25 years at recruitment, and (3) observational study design. Studies were excluded if they met the following criteria: (1) pain due to a known disease process (e.g., cancer), (2) pain not characterized as chronic, (3) pain characterized as an adverse effect of psychostimulant medication, (4) ADHD was not defined or measured in the sample, (5) studies for which ADHD was an exclusion criteria, (6) studies of non-human animals, (7) studies in languages other than English or Swedish, (8) case-reports or small (*n* < 5) case series, and (9) studies on the association between headaches and ADHD were also excluded after recent systematic reviews on this topic were identified [35,36]. Chronic pain was defined as non-headache pain lasting >3 months through patient identification of chronic pain, or diagnosis of a chronic pain condition also met this criteria.

#### 2.1.2. Study Selection

**Figure 1:**
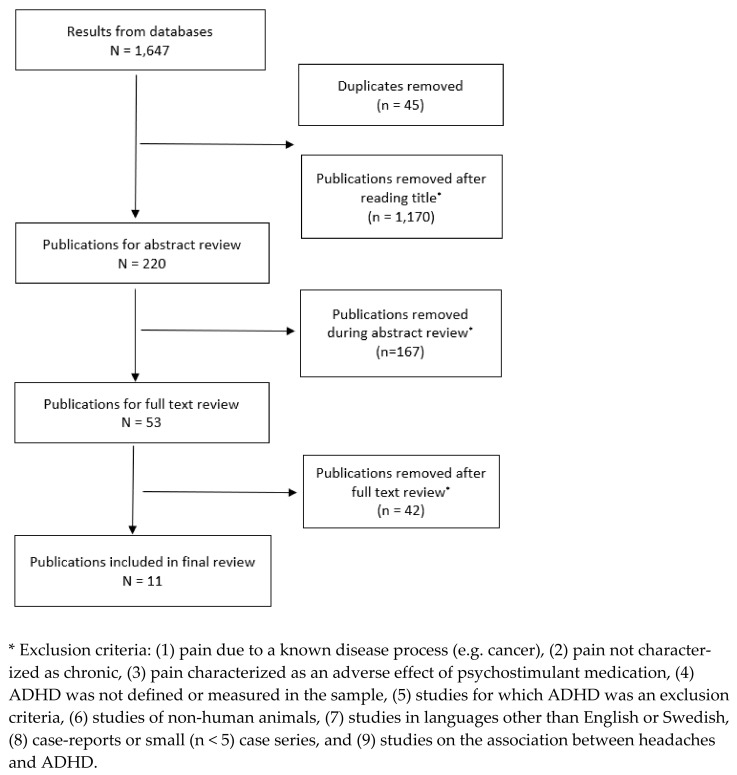
Study Selection.

The search strategy yielded 1647 results, from which 45 duplicates were removed. No additional articles were identified through reference searching. 1170 articles were excluded after reading the publication title. 167 were removed during abstract review, with 53 undergoing full text review. Following full-text review, 11 articles were selected for inclusion in the review (see Figure 1). 

Review of all abstracts was performed by one author (PB), and a second author (EB) was consulted when it was not immediately clear whether an article met study criteria or was related to the research question. Full review of the remaining papers was performed by two authors (EB, PB). All disagreements were referred to a third author (AW). 

### 2.2. Results

#### 2.2.1. Characteristics of Included Studies

Table 1 describes the objectives, approach and key results of the 11 included studies. Publication dates were between 1993 and 2021. Study designs included cross-sectional studies, cohort studies, case-control studies and case series. Mean participant ages were 8–15 years, and studies originated from six different countries. 

#### 2.2.2. ADHD Prevalence in Samples of Youth with Chronic Pain

Eight studies reported on the prevalence of ADHD in youth with chronic pain, with estimates ranging between 15 and 25%. Lipsker et al. [47] reported a prevalence of 19.9% (*n* = 126) using the Conners-3 parent report, and Low Kapalu et al. [48] found a prevalence of 18% (*n* = 94) based on a neuropsychological battery. Similar rates were found in smaller samples; Woodbury et al. [51] found a prevalence of 18% (*n* = 50) of children with functional abdominal pain using clinical interviews and the Conners-3 rating scale. Ghanizadeh et al. [45] used the semi-structured Schedule for Affective Disorders and Schizophrenia for School Age Children (K-SADS) and described a prevalence of ADHD in 15.6% (*n* = 45) of youth in a similar pain sample of functional abdominal pain compared to controls (0%; *n* = 45). Conversely, Galli et al. [44] did not find significantly higher rates of ADHD among youth with abdominal pain when compared with healthy controls. Of note, this study utilized only the CBCL checklist as a measure of ADHD, which is a less rigorous diagnostic mechanism than other studies (see Table 1). In a study of 17 female youth with Complex Regional Pain Syndrome (CRPS) by Cruz et al. [43], 23.5% had previously diagnosed ADHD, and neuropsychological testing revealed that 36% of the participants were at risk for poor attention and working memory deficits. However, only 11 participants completed the study tasks. A study of youth diagnosed with Developmental Coordination Disorder (DCD) found that children who met criteria for DCD and Ehlers–Danlos syndrome (Joint Hypermobility Syndrome; *n* = 19) were significantly more likely to meet criteria for ADHD than the youth in the DCD-only (*n* = 22) group (89% vs. 36%) [42]. Similarly, in a review of children with Ehlers–Danlos Syndrome/Joint Hypermobility Syndrome (*n* = 201), high rates of ADHD diagnoses based on chart reviews were identified in the sample, ranging from 11% to 46% (increasing with age) [46]. Compared to prevalence rates in large epidemiological studies [13,14], these studies show higher rates of ADHD among youth with chronic pain compared to the general population. Studies included in this review varied widely in the measurement approaches for assessing ADHD and chronic pain. For instance, ADHD diagnoses were determined through a variety of validated self- and proxy-report measures, ICD-code chart review, or comprehensive psychological evaluations. Similarly, the presence of chronic pain was identified through ICD-codes, questionnaires, or comprehensive medical evaluations (see Table 1). 

#### 2.2.3. Chronic Pain Prevalence in Samples of Youth with ADHD

Two studies identified in the current review that recruited from psychiatric samples described the prevalence of chronic pain in youth with ADHD. Mangerud et al. [49] reported that 65.9% (*n* = 216) of a sample of adolescents referred for ADHD (using ICD-10 codes) met chronic pain criteria (at least weekly pain lasting >3 months). In a second study, Asztely et al. [30] (*n* = 74) found that women who were recruited and diagnosed with ADHD between ages 3 and 18 years (using diagnostic semi-structured interviews) reported a high prevalence of chronic pain (>3 months of musculoskeletal pain, headaches or abdominal pain) in adulthood (M age = 27). Compared to prevalence rates in the general population in epidemiological research [13,14], these studies show higher rates of chronic pain than in individuals with ADHD, suggesting there may be higher pain prevalence in individuals with ADHD.

Asztély et al. also measured the effect of stimulants in their sample. They found lower rates of chronic pain among those treated with stimulants. However, the small sample size and lack of controls in the study design are limitations. Conversely, Wolff et al. [50] found that youth with ADHD had reduced pain perception. However, the effect disappeared if youth with ADHD were treated with stimulant medication. These studies offer very preliminary evidence that the use of stimulant medications may influence chronic pain experiences in youth with ADHD.

#### 2.2.4. Additional Relevant Studies of Pain Symptomatology and ADHD That Did Not Meet Identified Criteria for Standard Definitions of Chronic Pain 

In addition to the ADHD and chronic pain prevalence studies reviewed above, our review identified a number of studies that were of interest but did not meet the inclusion criteria for chronic pain as described above (e.g., did not specify pain duration >3 months). Significant associations have been found between ADHD and experiences of abdominal pain [52], physical complaints [53] and stomach aches [54]. However, a study with 474 children with ADHD seen at psychiatric clinics found that 29.1% reported clinically significant pain in the past month, but that the severity of ADHD symptoms was not associated with pain intensity [55].

Studies with case control designs and non-inclusionary pain presentations have mixed findings. Nationwide claims data in Germany [56] revealed that children with ADHD (*n* = 258,662) were more likely to be diagnosed with several somatic diseases compared to matched controls, including musculoskeletal system and connective tissue diagnoses (e.g., auto-inflammatory syndromes). Meanwhile, 165 children in Canada with ADHD reported bodily pain/discomfort that did not differ significantly from the standardized population mean [57]. Similarly, 76 youth with non-cardiac chest pain reported no difference of ADHD symptoms compared to controls [58].

Although these additional studies are heterogeneous and comparisons between them cannot easily be made, the overall findings are consistent with studies focused on ADHD and chronic pain that met our definition for inclusion, such as findings from Asztely et al.’s work. These findings suggest that ADHD is associated with a wide range of pain symptomatology. 

## 3. Aim 2: Potential Associations between Chronic Pain and ADHD over Time

**Figure 2:**
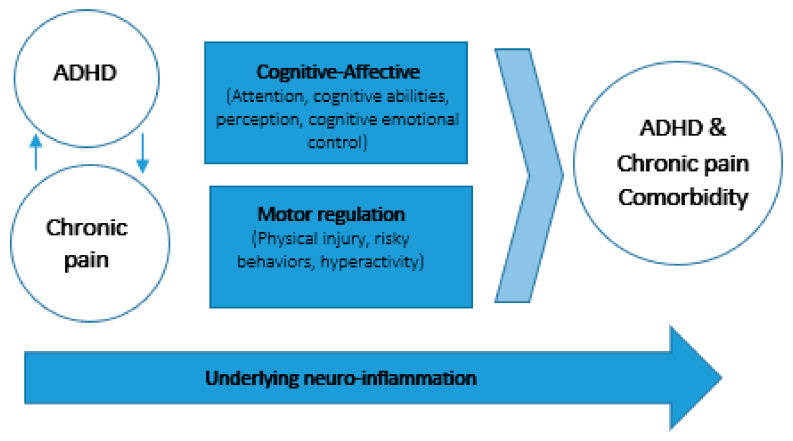
Potential associations between chronic pain and ADHD over time.

We suggest two potential co-occurring factors (cognitive–affective and motor regulation) that influence pain and ADHD over time. We also propose one potential underlying shared mechanism (neuroinflammation) that may influence the association between ADHD and chronic pain and comorbidity (See Figure 2). 

### 3.1. Cognitive–Affective Models of Pain Highlight the Role of Attention

The relationship between cognitive function and the pain experience is important since attention appears to play a major role in pain processing. Cognitive-affective models of pain suggest that attentional processes are negatively impacted by pain and that pain might affect brain regions related to cognition [59,60]. Research across multiple chronic pain conditions in adults suggests that persistent pain might contribute to diminished cognitive abilities, which can change perceptive and interoceptive states and alter inhibition of top–down cognitive control of emotional states [59]. Research with youth demonstrate similar patterns, where impaired attention is demonstrated in a range of pediatric chronic pain conditions [61,62].

### 3.2. Motor Regulation, Injury and Pain

Motor regulation problems are common among youth with ADHD [63,64,65] and may increase risk for physical injury across the lifespan [66,67]. ADHD also appears to increase risk for motor regulation problems and pain experiences in adulthood [26,68]. Research suggests that the underlying mechanism for this pattern involves ADHD symptoms (specifically inattention, hyperactivity and impulsivity), the role of comorbid psychiatric disorders (e.g., conduct disorder, oppositional defiance disorder, depression), risky driving behaviors, and parental risk factors (permissive parenting style, parent diagnosis of ADHD) [67]. Similar motor problems have been observed in youth with juvenile fibromyalgia [69].

### 3.3. Neuroinflammation

Considerable evidence points to the central role of neuroinflammation in the pathophysiology of both chronic pain and ADHD. Its role in pain has been demonstrated primarily through mechanisms related to central sensitization, pro-inflammatory cytokines, and impairment to the dopaminergic system [70,71]. Research on ADHD points to neuroinflammatory mechanisms as risk factors for developing ADHD [72,73]. Central components of neuroinflammation relevant to ADHD include glial activation, increased oxidative stress and neurotransmitter metabolism [74]. Kerkes et al. [15] suggest that in individuals with ADHD, central neuroinflammation underlies altered pain perception and pain sensitization. 

## 4. Discussion and Future Directions

This review sought to examine the potential association between ADHD and pediatric pain and provide a scoping review of the current literature. The findings from the scoping review reveal several important considerations. First, the quality of the studies in this review varied in regard to sample size, design (i.e., cross-sectional, case control), and measurement approaches for assessing pain and ADHD. Despite heterogeneity between studies, the majority of findings suggest there is an association between chronic pain and ADHD with comorbidity of both conditions significantly above average population prevalence. However, assessment of comorbid chronic non-headache pain in youth with ADHD has received little attention in the literature. Furthermore, limitations of this review ought to be noted, such as the inclusion bias for prevalence, as there might be unpublished work with null findings. Additionally, most samples in these studies are overwhelmingly white and from industrialized countries. 

Secondly, despite the potential mechanisms for the association between chronic pain and ADHD, not many studies tested these theories. For example, comprehensive neuropsychological testing used in work by Low Kapalu et al. and Cruz et al. suggest that evidence of cognitive deficits can be found in a chronic pain population, but the methodology of the studies does not offer enough evidence to support the cognitive–affective model. Moreover, assessed stimulant use [30] reported cross-sectional data only, without controlled methods to assess the possible mechanisms of stimulant use effects on pain. However, Wolff et al. [50] did include control groups and suggest that ADHD reduces pain perception, leading to higher risk tolerance and higher risk behaviors. They posit that since the effect disappears for youth with ADHD on stimulant medication treatment, the mechanism is possibly attributable to differential dopamine release. Although the study utilized controls, it was not a randomized design. Furthermore, no studies were found that used objective measures (e.g., fMRI, biomarkers of inflammation) that could elucidate the hypothesis of central neuroinflammation. 

Third, due to the lack of prospective, longitudinal and controlled research with large samples, the potential for ADHD as a potential risk factor for chronic pain over time is not well understood. The majority of the studies rely on retrospective or cross-sectional reports where temporal inferences cannot be made. Moreover, sex differences among youth with chronic pain and ADHD have not been addressed. While females are at higher risk for chronic pain, and males are at higher risk for ADHD, the sex differences for both are complicated and not yet well understood [75,76,77]. It is crucial to investigate how sex differences as differential risk/protective factors might intersect with the relationship between chronic pain and ADHD. 

Based on the findings in this review, we propose several considerations for next steps and future research. We suggest future work implements research designs that carefully assess for ADHD diagnoses and chronic pain onset and uses matched controls. These designs need robust and well-established diagnostic criteria for chronic pain and ADHD. Furthermore, for a more robust assessment of chronic pain and its effects, it would be important to look at pain-related impairment as well. Other psychiatric comorbidities (e.g., depression, anxiety) that affect chronic pain should be integrated to the model assessing for the interactions of ADHD and chronic pain. As previous research has shown, interaction effects of psychiatric comorbidities are present in both chronic pain and ADHD research [8,17]. Moreover, it is essential to examine whether the models proposed in this review are generalizable across chronic pain conditions or if specific chronic pain conditions need to include specific mechanisms. For example, a recent model by Baeza-Velasco [78] offers a framework for several neurodevelopmental disorders and joint hypermobility, hypermobility spectrum disorders, and Ehlers–Danlos syndromes. It highlights hypo- and hyperactivity symptoms and how they might play a role in these chronic pain conditions. It is unknown if this conceptualization is relevant to other chronic pain experiences. Therefore, more research is needed to determine if there are differential effects in associations of ADHD symptoms and chronic pain. Finally, future research could aid in screening youth and identifying screening approaches if ADHD is found to increase risk for chronic pain. Findings can lead to the development of early intervention for both ADHD and pain.

## Figures and Tables

**Table 1:** Overview of studies included in review.

Author, Year, Country	Aims	Study Design	Study Population	Measured Variables (ADHD; Pain)	Main Findings
Asztely et al. (2019); Sweden [30]	To investigate the prevalence of chronic pain in women with a diagnosis of ADHD in childhood.	16–19 year follow-up of cohort of 100 females who were initially referred to a neuropsychiatric clinic; 46% of participants had ADHD at the baseline assessment. Analysis was limited to those not lost to follow-up with a primary or secondary diagnosis of ADHD (*n* = 74).	74 females aged 3–18 at time of ADHD diagnosis. Follow-up was conducted at ages 19–37 (Mage = 27.2, SD= 4.2).	**ADHD:** Semi-structured interview by an experienced clinician based on DSM-IV criteria. **Pain:** Chronic widespread pain (CWP): >3 months of musculoskeletal pain, above and below the waist, on both sides of the body and involving the axial skeleton; >3 months of pain in the head or abdominal regions.	- Participants with a primary diagnosis of ADHD (*n* = 41), 39.0% reported CWP at follow-up. 31.7% reported chronic abdominal pain and 31.7% reported chronic headache. - Participants with ADHD treated with stimulants (24/74, 32.4%) had a statistically significantly lower prevalence of CWP than those with untreated ADHD (16.7% vs. 42.0%).
Celletti et al. (2015); Italy [42]	To assess the prevalence of generalized joint hypermobility (gJHM) in a group of 41 Italian children with development coordination disorder (DCD).	Compared groups of children with DCD who did and did not meet criteria for gJHM. Linear regressions were performed to assess for associations with ADHD.	41 children (Mage = 8; 31 males, 10 females) from specialty clinics.	**ADHD:** Assessed using DSM-IV criteria by investigators. **Pain:** Physicians used Beighton scores to diagnose generalized Joint Hypermobility Syndrome, questionnaires asked about symptoms related to gJHM.	- Statistically significant higher proportion of gJHM group with frequent falls, bruising and prolonged bleeding, motor impersistence, arthralgias and myalgias, intestinal constipation, sore hands from writing, and ADHD. - Beighton score was positively associated with ADHD.
Cruz et al. (2011); USA [43]	To describe neuropsychological profiles of children with Type 1 Complex Regional Pain Syndrome (CRPS-I) receiving inpatient rehabilitation.	Children admitted to an inpatient rehabilitation facility completed comprehensive neuropsychological assessments. Performances on neuropsychological measures were compared with age-based norms.	17 female children ages 9–18 (mean not calculated) with CRPS-I admitted to a neurorehabilitation unit.	**ADHD:** Attention problems subscale of the parent report version of Behavior Assessment System for Children (BASC) and BASC-2, prior ADHD diagnosis, Continuous Performance Test (CPT-II), Brief Test of Attention. **Pain:** CRPS-I diagnosis by physician, Numerical Rating Scale (NRS; 0–10).	- 4 of 17 patients (23.5%) had previously diagnosed ADHD. - Mean BASC score was within normal limits. - Overall mean standard score on the Brief Test of Attention was at-risk/impaired (M = 82.67, SD = 15.19, *n* = 6).
Galli et al. (2006); Italy [44]	To compare the prevalence of internalizing and externalizing disorders between children with recurrent abdominal pain (RAP), headache, and a control group.	Case-controlled study. ANOVA one-way analysis was used to compare Child Behavior Checklist 4–18 (CBCL) scales and subscales between groups.	70 children with recurrent abdominal pain (Mage = 9, SD = 3.6) and 70 healthy controls (Mage = 11.6, SD = 4.6).	**ADHD:** CBCL, parent-report. **Pain:** Recurrent Abdominal Pain (RAP), clinical diagnosis according to Rome II criteria.	ANOVA showed a highly statistically significant difference between attention for children with RAP (4.03), headache (5.06) and controls (3.27), but pairwise differences between children with RAP and healthy controls were not significant.
Ghanizadeh et al. (2008); Iran [45]	To compare the prevalence of psychiatric disorders between youth with functional abdominal pain syndrome (FAPS), with organic abdominal pain, and without chronic pain.	Case-controlled study over 2 years at a pediatric gastroenterology clinic. Psychiatrists performed blinded interviews and ANOVAs were performed to compare groups.	45 children with FAPS (ages 5–18, Mage = 10.8, SD = 3.6), 45 children with organic abdominal pain (ages 5–18, Mage = 11.8, SD = 3.3 children pain) and pain-free children (ages 5–18, Mage = 11.3, SD = 3.6).	**ADHD:** Semi-structured interview using the Kiddie Schedule for Affective Disorders and Schizophrenia (K-SADS). **Pain:** Functional abdominal pain syndrome (FAPS), clinical diagnosis according to Rome III criteria.	Prevalence of ADHD differed significantly among children with FAPS (15.6%), organic abdominal pain (2.2%), and pain-free controls (0%).
Kindgren et al. (2021); Sweden [46]	To establish the prevalence of ADHD and ASD among children with Hypermobility Spectrum Disorders (HSD) and hypermobile Ehlers–Danlos Syndrome (hEDS).	Retrospective study of children in the clinic since 2012 who received treatment for HSD or EDS. Chi-square and t-tests were used to compare groups.	201 children ages 6–18 (Mage = 12); 113 females, 88 males treated at one pediatric and youth medicine clinic with diagnoses of HDS or hEDS.	**ADHD:** Prior diagnosis in medical record. **Pain:** Prior ICD-10 diagnosis of HDS or hEDS in medical record. Beighton hypermobility scale in medical record.	16% of all subjects had a verified ADHD diagnosis and a further 7% were undergoing ADHD diagnostic investigation. Significantly more children with hEDS had ADHD compared to children with HSD. -
Lipsker et al. (2018); Sweden [47]	To examine (1) the clinically significant traits and symptoms of ASD and ADHD, (2) the proportion with previously diagnosed ADHD or ASD, and (3) differences in demographic and pain-related variables between the two groups.	Cross-sectional study examining parent–child dyads referred to a tertiary pain clinic.	126 children and adolescents (ages = 8–17, M = 14.6) (70% female) consecutively referred for chronic pain to a specialized pain clinic.	**ADHD:** Conners-3 parent report **Pain:** Children completed the Lübeck Pain Questionnaire (LPQ). Co-occurrence of other chronic somatic disease is assessed in the LPQ using an open-ended write-in option. Intensity of pain was assessed with a visual analog scale (VAS).	Mean ADHD T-score was 55.42 (vs. population mean of 50) with 19.9% scoring as “clinically significant” (T ≥ 65).
Low Kapalu et al. (2018); USA [48]	To examine the cognitive or neuropsychological functioning of youth with chronic pain.	Case series of youth entering an intense interdisciplinary pain treatment program.	94 youth with chronic pain (85.1% female, ages 10–18, Mage = 15.41, SD = 1.96) who were beginningintensive interdisciplinary pain treatment (IIPT).	**ADHD:** Wechsler Abbreviated Scale of Intelligence-II, Wechsler Intelligence Scale for Children-II; Processing Speed subtest (PSI), Grooved Pegboard Test, California Verbal Learning Test, Rey Complex Figure Test, WRAT-4, Conners Continuous Performance Test-II, Behavior Rating Inventory of Executive Functioning. **Pain:** 100 mm Visual Analogue Scale for pain.	-18% of participants had ADHD. -Youth with chronic pain had higher verbal comprehension and full-scale IQ scores than expected, below-average non-dominant hand dexterity, and difficulty with visual recall. -Performance on neuropsychological measures were generally not associated with pain intensity.
Mangerud et al. (2013); Norway [49]	To investigate frequency and location of chronic pain and pain-related disability among adolescent patients presenting to a psychiatric clinic.	Cross-sectional study that was part of a lager cohort study at a department of child and adolescent psychiatry.	717 children seen by a Department of Child and Adolescent Psychiatry (43.5% of those eligible to participate, ages 13–18, Mage = 15.7, SD = 1.7). 30.1% had ADHD as their primary diagnosis.	**ADHD:** ICD-10 diagnosis of ADHD as the reason for presentation to the clinic. **Pain:** Chronic pain defined as weekly pain over the last 3 months not related to known disease or injury. Pain was considered musculoskeletal if present in the neck, shoulder, back, buttocks, chest or extremities. Pain in ≥3 sites was considered multi-site pain.	Prevalence of chronic pain was 65.9% among adolescents with ADHD, with 55.1% reporting musculoskeletal pain and 31.3% reporting multi-site pain.
Wolff et al. (2016); Germany [50]	To examine pain perception in children and adolescents with ADHD and the interaction between pain perception and the administration of methylphenidate (MPH).	Sample from “German Health Interview and Examination Survey for Children and Adolescents” (KiGGS) cohort. Examined parents’ assessments of children’s pain distribution and pain perception as well as differences in pain perception between subjects receiving and not receiving MPH.	260 children and adolescents ages 3–17; 184 male, 76 female. 50% healthy controls, 25% ADHD not on MPH, 25% ADHD on MPH.	**ADHD:** Parent report of prior ADHD diagnosis (validated by Strengths and Difficulties Questionnaire (SDQ) Hyperactivity scale). **Pain:** Retrospective pain rating for past 3 month. Parent report for children aged 7–10 and self-report for children aged 11–17.	Children and adolescents diagnosed with ADHD without MPH treatment had significantly lower pain ratings compared to both healthy controls (HC) and those with ADHD treated with MPH.
Woodbury (1993); USA [51]	To investigate stressors, psychopathology, and biopsychosocial treatment of children with recurrent abdominal pain (RAP).	50 children were referred to the authors with non-organic RAP, with persistent dysfunction despite diet changes.	50 children (58% male, ages 4–20, Mage = 8) with nonorganic recurrent abdominal pain.	**ADHD:** Clinical interview based on the DSM-III, Conners-Teacher version. Unspecified psychological testing and/or neuropsychological testing completed for children presenting with learning problems only. **Pain:** Met clinical criteria for RAP	18% of participants had ADHD.

## Data Availability

The data presented in this manuscript can be provided by the corresponding author upon reasonable request.

## Supplementary Materials

The following supporting information can be downloaded at: https://www.mdpi.com/article/10.3390/children10010142/s1.
