# Supplementary material for: Associations between Chronic Pain and Attention-Deficit Hyperactivity Disorder (ADHD) in Youth: A Scoping Review
Source: Children (Basel). 2023 Jan 11;10(1):142. doi: 10.3390/children10010142 (PMC9857366; doi:10.3390/children10010142)
Supplement: Supplementary file 1 [file children-10-00142-s001.zip › children-2108403-supplementary.pdf]

Supplementary Materials

Using the Ovid search engine, MEDLINE, PsycINFO, and Cochrane Database of Systematic Reviews data bases were searched for terms related to attention impairment, children or adolescents, and pain or pain-related health problems. The following search query was used:

"Attention Deficit and Disruptive Behavior Disorder"/ or "Attention Deficit Disorders with Hyperactivity"/ or Hyperkinesis/ or "Hyperkinetic Syndrome"/ or "Minimal Brain Dysfunction"/ or (ADHD* or ADDH or ADHS or ADSH or "AD/HD" or HKD or MBD or (Attention* adj2 (Deficit* or impair* or problem*)) or "attention-deficit*" or (("Minimal Cerebral" or "Minimal Brain") adj2 (damage* or dyfunc* or disorder*)) or ((hyperkine* or overactive* or hyperactiv* or "hyper-activ*") adj2 (syndrome* or disorder*))).tw. or ("ASRS-Adolescent" or "SWAN" or "AIM-C" or "DAYAS*" or "Swanson, Kotkin, Agler, M-Flynn, and Pelham Scale" or "SKAMP" or "Swanson, Nolan, and Pelham Rating Scale" or "SNAP-IV*" or "SNAP-V*" or "VADRS" or "Conners Rating Scale*" or "Conners 3" or "Conners-Wells" or "Weiss Functional Impairment Rating Scale" or "WFIRS" or (("Child Behavior Checklist" or "CBCL") and "Attention Problem*") or (("K-SADS*" or "KSADS*" or "Kiddie Schedule for Affective Disorders and Schizophrenia") and "attention") or ("C-DISC" and "attention")).tw.
AND
Adolescent/ or "Adolescent Behavior"/ or "Adolescent Development"/ or "Adolescent Health"/ or "Adolescent Health Services"/ or "Adolescent Medicine"/ or "Adolescent Psychiatry"/ or "Adolescent, Hospitalized"/ or "Adolescent, Institutionalized"/ or "National Longitudinal Study of Adolescent Health"/ or "Psychology, Adolescent"/ or exp Child/ or "Child Behavior"/ or "Problem Behavior"/ or "Child Development"/ or exp "Child, Exceptional"/ or "Child Health"/ or "Child Health Services"/ or "Child Psychiatry"/ or "Child Rearing"/ or "Child, Hospitalized"/ or "Child, Institutionalized"/ or "Early Intervention, Educational"/ or "National Institute of Child Health and Human Development (U.S.)"/ or "Psychology, Child"/ or Minors/ or Puberty/ or Pediatrics/ or Schools/ or "Schools, Nursery"/ or "Young Adult"/ or ("elementary school*" or highschool* or "high-school*" or "high school*" or kindergar* or "nursery school*" or preschool* or "pre-school*" or "primary school*" or schoolage* or "school-age*" or "school age*" or schoolboy* or "school-boy*" or schoolchild* or "school-child*" or schoolgirl* or "school-girl*" or "secondary school*" or "college student*" or "university student*").tw. or (adoles* or boy* or child* or girl* or kid or kids or pediatric* or paediatric* or preteen* or "pre-teen*" or pubert* or pubescen* or teen* or toddler* or "young people" or "young person" or youth*).tw.
AND
exp Pain/ or "Pain Clinics"/ or "Pain Management"/ or exp "Complex Regional Pain Syndromes"/ or "Fibromyalgia"/ or "Patellofemoral Pain Syndrome"/ or "Temporomandibular Joint Dysfunction Syndrome"/ or ("pain" or "pains" or "pained" or "paining" or "painful*").tw. or ("Central Sensitivity Syndrome*" or colic* or erythromelalg* or "Failed Back Surgery Syndrome*" or fibromyalg* or "Interstitial Cystit*" or "Morton Neuroma*" or "Phantom Limb*" or "Piriformis Muscle Syndrome*" or "Reflex Sympathetic Dystroph*" or sciatica or "Slit Ventricle Syndrome*" or "Symphysis Pubis Dysfunction" or (temporomandibular adj2 dysfunc*) or vaginismus or vulvodyn*).tw. or (abdominalg* or acrostealg* or adenalg* or alge* or algi* or algol* or algom* or angina or anginal or arthralg* or arthritis* or causalgia* or cephalg* or dyspeps* or dysmenorrhe* or dysur* or earache* or enterodyn* or esophagodyn* or gastralg* or glossalg* or hyperalges* or mastodyn* or metatarsalg* or mastalg* or myalg* or neuralg* or neuropath* or nocicept* or odontalg* or omalg* or ostalg* or otalg* or prosopalg* or rachialg* or toothache* or urethrodyn*).tw.
